# A Practical Treatise on the Diseases of Children

**Published:** 1846-10

**Authors:** 


### Art. XV.

*A Practical Treatise on the Diseases of Children.* By James Milman Coley, m.d., Member of the Royal College of Physicians in London, &c. —*London,* 1846. 8vo, pp. 467.

We regret much that we cannot speak of this work at all in terms of commendation; and we confess we cannot see why it was written, or, at least, why it has been published. The preface, it will be seen, is one of considerable pretensions, both with reference to the author and his book; but so far are we from thinking the book deserves to be regarded in the light in which Dr. Coley wishes to represent it—" as a comprehensive work of reference"—that we cannot give it even the credit due to a well-executed compilation.

The surgical diseases of childhood present, with the few exceptions of original malformations, no, or scarcely any, peculiarities requiring special mention, though, in his preface, the author takes much credit to himself for having noticed surgical diseases. And we regret to say that what he professes to do he has done ill. For example, he describes the operation for strangulated hernia, while all the very important questions as to the time when a truss should be applied, how you can best avoid the annoyance to a young infant inseparable from a truss becoming soiled with its discharges, the best form of truss, &c.—questions of real importance, since the age of the patient here really does modify our practice—are left unnoticed. Nothing is said about non-descent of the testicle, &c. &c.

At page 69, external hydrocephalus is used as a synonym for cephalhæmatoma! while this accident is noticed in the most cursory manner. The author speaks (p. 8) of *many* instances of success of the operation for imperfect anus, when such instances are notoriously very few, &c. &c. If from the chapters on diseases of the eye you take away all that is Mr. Lawrence's, and all that is Mr. Middlemore's, very little will remain for Dr. Coley. From the seventy pages on diseases of the skin we have learned nothing. In the preface the author glorifies himself for his notice of erysipelas. It is only during infancy that we find deviations from the ordinary course of erysipelas, such as—that it has a greater tendency to wander—that it often proves fatal in young children without either vesication or the formation of matter—that the former result is much rarer than the latter, &c. Dr. Coley notices none of these; and we find that the treatment recommended is what might be adopted for a man of 20 or 30. What guide would his directions be to a pupil who had to treat an infant of a month old? The important subjects of measles and scarlatina are slurred over in an extraordinary way; and his remarks on the dropsy after scarlatina are absurdly short.

But it is wearisome to go on noticing a book in which we can really find nothing to praise. Take, for instance, the chapter on gangrene of the mouth (p. 131). A page and a half are occupied with the translation of three post-mortem examinations by Rilliet and Barthez; the remaining two pages and a half are inferior to what might be expected from a second year's student. Immediately after these follows a notice of "Cancrum oris, sloughing phagedæna of the mouth;" in which the author appears to have confounded simple stomatitis with the affection he has described in the preceding section.

The article on dentition consists of four pages of common-place physiology. Then comes a scrap of French, which, as in many other instances, Dr. Coley leaves untranslated. Many little bits of French are thus introduced, mere scraps, often of no importance whatever (see p. 253). He denies that any other diseases than local inflammation are connected with dentition, and yet he does not notice at all that general inflammation of the gums of teething children, which any patient observer must often have noticed, and which has been described as Odontitis. No help is given towards fixing any rule about lancing the gums, though this is surely a matter on which, after forty years' practice, a man must have arrived at some conclusion.

There is no opinion given as to the advantage of the early operation for harelip (p. 152), though the subject has of late received much attention in France.

In his account of the diseases of the digestive organs, Billard, Rilliet, and Barthez have been drawn on very largely, but there is a popular physiology of his own very often introduced, which we do not at all like. See, for instance, the account of the anatomy and physiology of the digestive organs at pp. 165, 175. Whom is this meant to teach? It is certainly more adapted to the capacity of the monthly nurse than the medical practitioner, or even the most juvenile student.

The chapter on croup is, on the whole, a favorable specimen of the book; but what is there in it? It teaches nothing new, nor gives any weight to what was known before. In the chapter on pneumonia (p. 281) we have a description of all sorts of absurd and even impossible symptoms; the expectoration described in children who cannot spit, and delirium talked of in infants who have not yet attained the use of reason. The morbid appearances are entirely from Rilliet and Barthez, as is the case also in the article on phthisis. The important and extensive subject of diseases of the brain is compressed into the narrowest limits, and is as unsatisfactory as possible.

The general style of the work is slovenly, sometimes ungrammatical. Misprints abound, while there is a great attempt at a display of learning both in the text and notes. If, however, the reader will look at the wondrous authorities (German) quoted in the notes of pages 151, 250, 253, 264, &c., he will assuredly feel much doubt as to whether the author has ever seen the books he quotes, or could read them if he had.

Before concluding, we must say one word on the title-page of this book. We should be glad to be informed on what authority the author appends the M.D. to his name. We happen to know that he is not a graduate in medicine in any university or other institution qualified to confer the degree and title of "MEDICINÆ DOCTOR;" and we have always been taught to believe that a person who has not so graduated, is no more entitled to place after his name the two letters M.D., than he is to place the three letters KNT., or the four letters BART. The author of this volume is a LICENTIATE, or, as he writes it, a MEMBER of the London College of Physicians; and he is unquestionably legally qualified to practise as a physician; but the College neither has, nor pretends to have the power or authority, to grant to any one the degree of *Doctor in Medicine,* and therefore we regard the assumption of this title and dignity by a mere licentiate as altogether unwarrantable. As the licence of the College qualifies the holder, as we have said, to *practise* as a physician, it would not perhaps be overstraining the privilege, if such holder took *the name* as well as the office of physician. At least, we should ourselves not only readily accord this title, but we would even, out of courtesy, give the name of *Doctor* also, just as we give this name to Bachelors of Medicine. But just as no one ever saw a Bachelor of Medicine, however long he may have been *named* DR. in common parlance, place the talismanic letters M.D. after his name, so, *à fortiori,* no one ought ever to see an ungraduated licentiate of the College of Physicians to do so. Dr. Coley—as we are willing to style him by courtesy, though we believe his proper title to be Mr. Coley, Physician,—can certainly plead, in extenuation of his fault, that he is kept in countenance by many other of his fellow-licentiates, both *intra et extra urbem;* but it is principally on this very account that we notice the subject here, and we say—*peccatur intra muros et extra.* We know Dr. Coley to be a gentleman of the highest respectability; and though he has written a bad book, we believe him to be an experienced and skilful physician. No Doctor of Medicine, at whatsoever university he may have graduated, could hesitate for a moment to meet him in consultation; none, we should hope, would refuse to address him by the ordinary title; but it is well that the members of the profession generally, and the Fellows of the College of Physicians especially, should be made aware that there are to be found among the ungraduated licentiates of the college, men of a very different stamp, who are a disgrace to the title of *Doctor of Medicine,* which they so improperly assume—men who are not only utterly undisciplined in all that literary culture which a *degree* and its concomitant *title* are usually understood to imply, but who are even destitute of that amount of special knowledge which ought to be possessed by every member of the medical profession, however humble. Can such things be—and overcome us like a summer cloud, without our special wonder? But if any of our wondering readers should be curious also, and desire to know *how* such things be, we refer them for particulars to that famous statute which established the ELECTS of the College of Physicians.

